# Glucocorticoid‐remediable aldosteronism in a young adult with a family history of Conn's syndrome

**DOI:** 10.1002/ccr3.1377

**Published:** 2018-01-15

**Authors:** Heiko Methe, Sinan Pehlivanli

**Affiliations:** ^1^ Department of Cardiology Kliniken an der Paar Krankenhaus Aichach Aichach Germany

**Keywords:** Arterial hypertension, Conn's syndrome, glucocorticoid‐remediable aldosteronism

## Abstract

Glucocorticoid‐remediable aldosteronism is a hereditary form of primary hyperaldosteronism and the most common monogenic cause of hypertension. We present the case of a 24‐year‐old man with a family history of Conn's syndrome. Yet, in the index patient, classical characteristics of mineralocorticoid excess could be reversed by exogenous glucocorticoids.

## Introduction

Glucocorticoid‐remediable aldosteronism (GRA) is a rare form of hyperaldosteronism‐induced arterial hypertension. GRA is inherited as an autosomal dominant trait in which aldosterone synthesis is under control of adrenocorticotropic hormone. We report the case of a 24‐year‐old man with a diagnosis of GRA and a family history of Conn's syndrome.

Although most cases of arterial hypertension are essential, some cases have an identifiable cause. The most common form of secondary hypertension is primary aldosteronism. The two major subtypes of primary aldosteronism are bilateral idiopathic hyperaldosteronism (IHA, relative frequency 65%) and aldosterone‐producing adenoma (Conn's syndrome, 30%), whereas glucocorticoid‐remediable aldosteronism (GRA, familial hyperaldosteronism type I) accounts for <1% of cases of primary aldosteronism 1, 2.

Causes for IHA are unknown, whereas in some patients with Conn's syndrome, various mutations in the *KCNJ5* gene have been identified that regulate function of a potassium channel within the adrenal gland 3. These mutations result in less selectivity of the potassium channel allowing other ions –predominantly sodium –to pass as well, thereby resulting in increased aldosterone production. In contrast, the underlying genomic rearrangement for the development of GRA has been demonstrated by Lifton et al. 4, 5: GRA is caused by a chimeric gene duplication resulting from unequal crossing over between 11*β*‐hydroxylase (*CYP11B1*) and aldosterone synthase (*CYP11B2*) genes: The chimeric gene represents a fusion of the 5′ adrenocorticotropin‐responsive promotor region of the 11*β* ‐hydroxylase gene and the 3′ coding sequences of the aldosterone synthase gene. The characteristic clinical presentation was first summarized by Sutherland et al. 6. Steroid 11‐hydroxylase, which is regulated by ACTH, is normally only expressed in the zona fasciculata of the adrenal gland, whereas aldosterone synthase is normally only expressed in the zona glomerulosa. While Conn's syndrome and IHA are characterized by hyperplasia of one‐ or both‐sided zona glomerulosa, the gene duplication in GRA results in ectopic expression of aldosterone synthase activity in the cortisol‐producing zona fasciculata. GRA is inherited as an autosomal dominant trait. In contrast to Conn's syndrome, arterial hypertension in GRA typically manifests before the age of 21. GRA is a rare monogenic form of primary aldosteronism 7, 8; most cases being described in the USA 9, China 10, Japan 11, Italy 12, and few cases so far reported from Germany 13.

## Case Report

**Figure 1:**
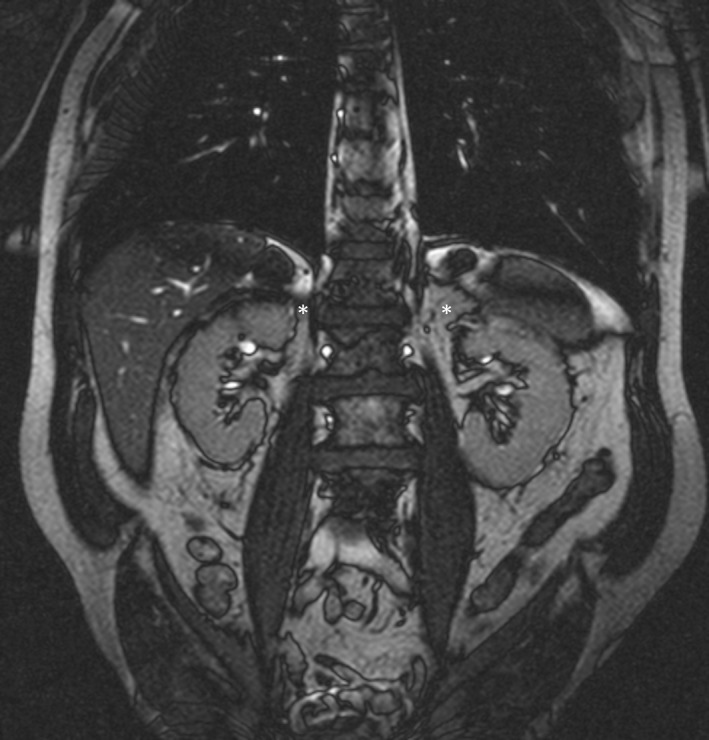
MRI patient. Abdominal MRI in the patient with glucocorticoid‐remediable aldosteronism did not show any abnormalities (adrenal glands marked with “_*_ *”*).

A 24‐year‐old male Caucasian was referred to our hospital for evaluation of secondary hypertension. He became symptomatic at the age of eleven. His blood pressure remained uncontrolled despite the treatment with four antihypertensive agents (AT1‐receptor antagonist, *β*‐blocker, calcium channel antagonist, and doxazosin mesylate). On initial examination, his blood pressure was 180/95 mmHg and his heart rate was 82 beats per minute. A 24‐ h ambulatory blood pressure measurement while on treatment revealed an average 24‐ h blood pressure of 183/98 mmHg with a heart rate of 71 bpm. There was no nocturnal dip. The remaining physical findings were normal. Serum electrolytes were as follows: sodium 139 mmol/L, potassium 4.4 mmol/L, calcium 2.7 mmol/L, anorganic phosphorus 4.1 mg/dL, magnesium 1.97 mg/dL. Renal, liver, and thyroid tests were all normal. 24‐h urine analysis showed normal values for catecholamines and catecholamine metabolites. Plasma renin activity under medication was undetectable (<1 ng/L), and plasma aldosterone concentration was moderately increased (237 ng/L), aldosterone urine excretion was within normal limits (0.44 *μ*g/day). MRI revealed no adrenal tumor and no other abnormalities **(**Fig. 1). Dexamethasone suppression testing (1.5 mg/day for 5 days) resulted in normalization of aldosterone plasma concentration (132 ng/L) and reduction in urine excretion of 18‐hydroxycorticosterone (31–4.2 *μ*g/24 h) and 18‐hydroxycortisol (1760–110 *μ*g/24 h). Upon posture testing, plasma aldosterone concentration rose to 304 ng/L, an increase by 28%. Polymerase chain reaction (PCR) amplification and analysis of the resulting fragments were performed as previously described 14. The patient's deoxyribonucleic acid (DNA) produced the characteristic 3.9‐kb product when the 5′ primer for 11*β* ‐hydroxlase and the 3′‐primer for aldosterone synthase were used for amplification.

The patient refused long‐term treatment with dexamethasone and was placed on an aldosterone antagonist resulting in marked improvement of blood pressure with reduction in antihypertensive drugs: In the follow‐up course of twelve months, he had physiologic systolic and diastolic blood pressure while being treated with spironolactone, AT1‐receptor antagonist, and calcium antagonist.

**Figure 2:**
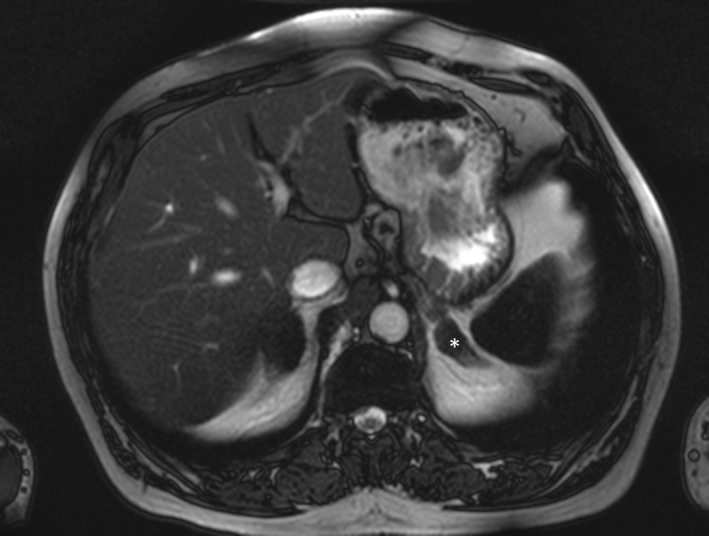
MRI father. Abdominal MRI of the patient's father revealed a nodule in the left adrenal gland (“_*_”).

Three years before, the patients’ blood‐related father was diagnosed with Conn's syndrome in another hospital. He had had developed arterial hypertension at the age of 45. His blood pressure was 150/86 mmHg on triple treatment (AT1‐receptor antagonist, *β*‐blocker, and calcium channel antagonist). Serum potassium level was 2.9 mmol/L, sodium 147 mmol/L. He had mild metabolic alkalosis. Plasma renin activity under medication was undetectable (<1 ng/L), and plasma aldosterone concentration was markedly increased (732 ng/L). Plasma levels of ACTH, cortisol and catecholamines, and urinary excretion of free cortisol and catecholamines were within normal range. Plasma rennin activity remained suppressed and plasma aldosterone concentration was 714 ng/L after furosemide infusion plus upright test, indicating autonomous secretion of aldosterone. An abdominal MRI scan showed a nodule in the left adrenal gland. The right adrenal gland showed no obvious nodules (Fig. 2). Selective adrenal vein sampling demonstrated a threefold increase in aldosterone concentration in the left adrenal vein over the right adrenal vein. Laparoscopic total left adrenalectomy was performed, and 2 weeks after surgery, potassium levels and blood pressure levels were within normal range upon therapy with an AT1 receptor antagonist and a calcium channel blocker. PCR amplification and analysis did not show the typical 3.9‐kb fragment indicating the presence of the chimeric *CYP11B1/CYP11B2* gene in GRA patient's genome.

Yet, PCR analysis in the blood‐related mother revealed that she was affected by the exact chimeric gene as was found in our patient without clinical manifestation of arterial hypertension. No differences in results on PCR analysis could be observed between our patient's and his mother's results. This is in line with other reports demonstrating that expressivity and penetrance of the GRA gene can be variable and that the presence of GRA gene mutation does not necessarily predict the clinical phenotype of hyperaldosteronism 15, 16, 17.

The motherly aunt of our patient died from hemorrhagic stroke due to a ruptured intracranial aneurysm at the age of 24. No genetic testing could be performed. No further family member (older sister, younger brother) is affected by arterial hypertension or tested positive for this mutation.

## Discussion

We report here a case of arterial hypertension due to GRA in a 24‐year‐old patient with a positive family history of Conn's syndrome.

Availability and wider application of the plasma aldosterone/renin ratio as a screening test for primary aldosteronism have led to the recognition that primary aldosteronism is the most common potentially curable and specifically treatable form of hypertension, possibly accounting for as many as 5–13% of patients. Causes for IHA are still unknown; in contrast, a minority of patients with adrenal adenoma have been identified to bear mutations in the *KCNJ5* gene 3. Yet, in early onset aldosteronism, genetic testing for the causative “hybrid” 11beta‐hydroxylase/aldosterone synthase gene identifies patients with GRA 14. GRA, also termed familial hyperaldosteronism type I, was first described in 1966 6. This autosomal dominant form of aldosteronism accounts for only 0.5–1% of primary aldosteronism: therefore, random genetic screening seems to be not advisable 18, 19.

It is well known that different sites of unequal crossover between the 5′‐regulatory ACTH‐responsive sequence of the *CYP11B1* gene and the 3′‐coding region of the *CYP11B2* gene might result in varying phenotypic effects 15 and that no relationship between genotype and phenotype has been found in GRA 16, 17. Furthermore, Fallo et al. speculated that differences in the phenotype might be attributed to altered regulation of the chimeric gene by specific inhibitors or repressors, or by other genes lying outside the locus of the chimeric gene 15.

Yet, our case is different and unique as within one generation of a single family, two different subtypes of aldosteronism appeared. On gene analysis, the normotensive mother was affected by a chimeric *CYP11B1/2* gene. Her sister died from a hemorrhagic stroke due to a ruptured intracranial aneurysm at the age of 24. Among patients with GRA, 18% had cerebrovascular complications including hemorrhagic strokes 20, 21.

Other reports demonstrated high prevalence of thoracoabdominal aneurysms in affected patients 22. Besides Conn's syndrome diagnosed and treated in the patient's father and the hemorrhagic stroke, the clinical family history of our patient is empty regarding arterial hypertension.

The present case sheds light on the importance to screen for secondary causes of arterial hypertension especially in young patients 23. Increased aldosterone/renin ratio followed by a structured work‐up strategy seems advisable 24. This should include imaging studies of the adrenal glands by CT/MRI scan, functional testing (urine excretion, posture test, dexamethasone suppression test), adrenal vein sampling and where appropriate genetic screening for GRA irrespectively of the family history 25, 26.

## Authorship

HM and SP: were involved in the medical care of the patient, participated in the acquisition and analysis of data, the literature review, and writing of the manuscript. Both authors approved the final version of the manuscript.

## Conflict of Interest

None declared.
